# Precision oncology for advanced oral cancer: synergizing multimodal therapy with dynamic biomarker monitoring

**DOI:** 10.1097/JS9.0000000000003012

**Published:** 2025-07-08

**Authors:** Jing Zhang, Gang Dong, Ting-Gang Fu

**Affiliations:** a Oral Prosthetics Teaching and Research Office, Qilu Medical University, Zibo, Shan dong, China; b Qilu Medical University,Zibo, Shan dong, China; c Department of Human Anatomy, Qilu Medical University, Zibo, Shan dong, China

We appreciate the authors’ work in the recent article “Neoadjuvant chemoimmunotherapy in resectable locally advanced oral squamous cell carcinoma: a single-center retrospective cohort study.” This article discusses therapeutic strategies for neoadjuvant therapy in resectable advanced oral squamous cell carcinoma (OSCC), providing practical guidance for optimizing OSCC treatment outcomes. Building upon this foundation, we offer the following insights for discussion^[1]^.

Although neoadjuvant therapy has been proven effective in reducing tumor size, downstaging disease, and significantly improving survival rates, the medical community generally advocates for a standardized integrated sequential approach combining systemic pharmacotherapy with radical surgery. This model comprises three critical phases: 1. Neoadjuvant Therapy (2–4 cycles): Administered preoperatively to shrink the primary tumor, enhance surgical feasibility/safety, and facilitate R0 resection. 2. Radical Surgery: Performed promptly after neoadjuvant therapy to achieve complete tumor resection (R0) and clear potential metastatic lymph nodes. 3. Adjuvant Consolidation Therapy: Postoperative treatment (e.g., chemoradiation or immunotherapy) to eliminate residual micrometastases/circulating tumor cells, thereby reducing recurrence and distant metastasis risks. This “neoadjuvant → surgery → adjuvant” triad demonstrates superior survival benefits compared to neoadjuvant therapy alone, as evidenced by recent trials (e.g., KEYNOTE-689, Optimal study) reporting 2-year overall survival (OS) rates exceeding 90% with combined strategies.

While PD-L1 expression (CPS ≥20), tumor mutational burden (TMB), and immune cell infiltration are established predictors for immune checkpoint inhibitors (ICIs) in recurrent/metastatic HNSCC, their utility in neoadjuvant settings for resectable OSCC remains limited. Key challenges include: 1. Tumor Heterogeneity: Spatial and dynamic variations in PD-L1 expression reduce its predictive reliability. 2. Immune Microenvironment Complexity: Single biomarkers (e.g., TMB) fail to fully capture immune response dynamics, yielding inconsistent results across studies. Emerging Solutions: Multimodal Biomarker Integration: Combining PD-L1, TMB, T-cell inflammation gene signatures, and microenvironment-specific immune subsets. Dynamic Monitoring: Tracking ctDNA clearance or tertiary lymphoid structure (TLS) density changes to refine patient selection^[2]^.

Platinum agents (e.g., cisplatin) induce high-emetogenic-risk (>90%) chemotherapy-induced nausea/vomiting (CINV), leading to dehydration, electrolyte imbalances, and treatment discontinuation^[3]^. The four-drug regimen is now recommended as first-line prophylaxis: Core Triplet: 5-HT_3_RA (e.g., ondansetron): Blocks acute vomiting via 5-HT_3_ receptors. NK-1RA (e.g., aprepitant): Targets delayed vomiting via substance P pathways. Dexamethasone: Potentiates antiemetic effects and reduces inflammation. Augmented Quadruplet: Adding olanzapine (a dopamine D_2_/5-HT_2_ C receptor antagonist) significantly improves control of refractory/delayed CINV and alleviates anxiety/insomnia, enhancing quality of life and treatment adherence.

Neoadjuvant therapy plays an irreplaceable role in OSCC management. Future studies should leverage multi-omics technologies (e.g., single-cell sequencing, microbiome analysis) to dissect tumor microenvironment dynamics, identify novel biomarkers (e.g., CD4⁺Tfh-CXCL13), and develop personalized predictive models for precision immunotherapy.

## Data Availability

Not applicable.
